# Mechanisms underlying the wound healing and tissue regeneration properties of a novel gauze dressing impregnated with traditional herbal medicine (Ya-Samarn-Phlae) in type 2 diabetic Goto-Kakizaki (GK) rats

**DOI:** 10.3389/fphar.2025.1574715

**Published:** 2025-04-09

**Authors:** Sineenart Sanpinit, Acharaporn Issuriya, Oraphan Sakulkeo, Palika Wetchakul, Surasak Limsuwan, Pinanong Na-Phatthalung, Siriwan Kantisin, Jian Tang, Sasitorn Chusri

**Affiliations:** ^1^ Department of Applied Thai Traditional Medicine, School of Medicine, Walailak University, Thasala, Nakhon Si Thammarat, Thailand; ^2^ Division of Health and Applied Sciences, Faculty of Science, Prince of Songkla University, Songkhla, Thailand; ^3^ Traditional Thai Medical Research and Innovation Center, Faculty of Traditional Thai Medicine, Prince of Songkla University, Hat Yai, Songkhla, Thailand; ^4^ Division of Hematology and Oncology, Icahn School of Medicine at Mount Sinai, New York, NY, United States; ^5^ Unit for Area-Based Research and Innovation in Cross-Border Health Care and Occupational Health and Safety Department, School of Health Science, Mae Fah Luang University, Chiang Rai, Thailand; ^6^ School of Chinese Medicine, Bozhou University, Bozhou, China; ^7^ School of Health Science and Biomedical Technology Research Group for Vulnerable Populations, Mae Fah Luang University, Chiang Rai, Thailand

**Keywords:** Ya-Samarn-Phlae, diabetic ulcers, wound healing, angiogenesis, traditional Thai medicine, chronic wounds, curcumin, α-mangostin

## Abstract

**Ethnopharmacological relevance:**

A traditional preparation of Ya-Samarn-Phlae (T-YaSP) consists of *Garcinia mangostana* L., *Oryza sativa* L., *Curcuma longa* L., and *Areca catechu* L. and has been used in Thai medicine as an infused oil for treating chronic and diabetic wounds. It is reputed for its antibacterial, antioxidant, and wound-healing properties. Despite its traditional use, scientific validation of the mechanisms underlying diabetic wound healing remains limited.

**Aim:**

This study aims to develop a novel gauze dressing impregnated with an ointment containing T-YaSP (YaSP) to enhance its practical application and elucidate the mechanisms of action in promoting wound healing in both non-diabetic and type 2 diabetic wounds of this ointment.

**Materials and methods:**

YaSP was developed and tested for stability and dermal irritation. Changes in chemical markers during storage were measured both qualitatively and quantitatively. Its anti-inflammatory activity was assessed using the carrageenan-induced rat paw edema model. The effect of YaSP on levels of nitric oxide (NO), myeloperoxidase (MPO), malondialdehyde (MDA), inflammatory cytokines (TNF-α, IL-1β, and PGE_2_), and pro-inflammatory enzymes (iNOS and COX-2) was measured. The wound-healing effects of YaSP were assessed using full-thickness (6 mm diameter) wound models in both non-diabetic Wistar rats and type 2 diabetic Goto-Kakizaki rats. In addition to evaluating wound closure on days 0, 3, 5, 7, 9, and 11, the influence on TGF-β1, VEGF, and the production of collagen types I and III, which indicate the inflammatory, proliferative, and remodeling phases, was measured.

**Results:**

During the 6-month storage period, the α-mangostin content measured in YaSP did not decrease; however, the curcumin level showed a significant reduction. Topical treatment with YaSP demonstrated strong anti-inflammatory activity and alleviated oxidative stress and inflammatory markers. YaSP improved wound closure rates in both diabetic and non-diabetic models. Levels of TGF-β1 and VEGF increased, indicating the promotion of angiogenesis and granulation tissue formation during the proliferation phase on the seventh day. Additionally, TGF-β1 levels dropped on the 11th day, aligning with diminished inflammation and enhanced remodeling. The treatment balanced collagen synthesis, increasing type III collagen in the early stages and type I collagen in the later stages of wound healing. Histological analysis confirmed reduced inflammation, enhanced neovascularization, and increased collagen production.

**Conclusion:**

A gauze dressing impregnated with YaSP provides a practical solution for diabetic wound management and demonstrates strong wound-healing properties by modulating excess inflammation, promoting angiogenesis during the proliferation phase, and regulating collagen synthesis throughout the remodeling phase. This discovery reveals, for the first time, the underlying mechanisms of action of this traditional formulation, highlighting its potential as a cost-effective alternative for managing chronic wounds in resource-limited settings.

## Graphical Abstract

**Figure:**
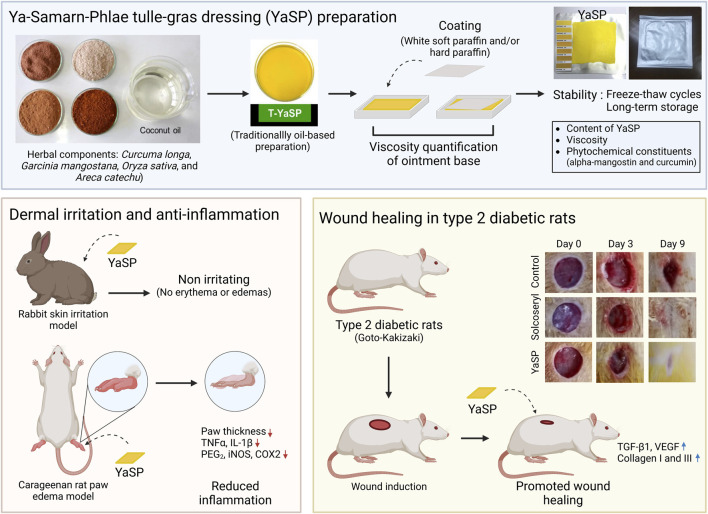

## 1 Introduction

The global prevalence of diabetes has risen sharply, from 200 million individuals in 1990 to an estimated 537 million in 2021, with projections reaching 643 million by 2030 and 783 million by 2045 (Sun et al., 2022). This alarming increase has brought about a parallel rise in diabetes-related complications, including diabetic foot ulcers (DFUs), which significantly impact patient morbidity and healthcare systems worldwide. The prevalence of DFUs is approximately 6% worldwide (Zhang et al., 2017), escalating to as much as 20%–30% in certain regions (Abdissa et al., 2021; International Diabetes Federation, 2022; Salad et al., 2023). In low-to middle-income countries (LMICs), where three out of four adults with diabetes live, DFUs pose significant challenges due to limited resources, inadequate healthcare infrastructure, delayed treatment, and insufficient prevention strategies (Kengne and Ramachandran, 2024; Lam et al., 2021). These ulcers frequently result in severe complications, including infections, amputations, and increased mortality risk (Ghadeer et al., 2025; Holman et al., 2024). Thus, effective management of DFUs is crucial for improving patient outcomes and reducing economic burdens.

Wound healing is a dynamic and intricate biological process encompassing several overlapping phases, including hemostasis, inflammation, proliferation, and tissue remodeling (Patel et al., 2019). In diabetic patients, the following factors, neuropathy, ischemia, and heightened susceptibility to infections, exacerbate the risk of chronic and infected wounds (Burgess et al., 2021; Patel et al., 2019). Hyperglycemia further impairs the healing process through multiple mechanisms, such as endothelial dysfunction, prolonged clotting time, and diminished growth factor secretion during the hemostasis phase. Chronic inflammation driven by elevated pro-inflammatory cytokines and oxidative stress, coupled with macrophage and neutrophil dysfunction, delays pathogen clearance and exacerbates tissue damage (Burgess et al., 2021; Patel et al., 2019; Worsley et al., 2023). Additionally, hyperglycemia disrupts the proliferative and remodeling phases by impairing keratinocyte migration, reducing vascular endothelial growth factor (VEGF) activity, and hindering fibroblast-mediated extracellular matrix (ECM) formation, ultimately compromising wound repair (Xu et al., 2024).

Despite advancements in standard care for DFUs, many wounds fail to heal effectively, underscoring the urgent need for alternative treatment approaches. Medicinal plants have emerged as promising candidates in this context, offering diverse biological activities that address the multifaceted challenges of diabetic wound healing (Ahmadian et al., 2021; Zamanifard et al., 2024). Well-established plants such as *Aloe vera* (Kania et al., 2024), *Centella asiatica* (Arribas-López et al., 2022), *Curcuma longa* (Chawla and Sharma, 2022), the emulsion containing allicin and quercetin (Nusantoro et al., 2024a), and *Camellia sinensis* (Xu et al., 2021) have demonstrated anti-infective, antioxidant, anti-inflammatory, and pro-regenerative properties. These plants reduce excessive inflammation, enhance collagen synthesis, stimulate angiogenesis, and promote keratinocyte proliferation, often providing cost-effective and accessible solutions compared to conventional treatments. However, challenges such as formulation development, variability in patient responsiveness, and limited clinical evidence hinder their widespread application, necessitating further research (Ahmadian et al., 2021; Herman and Herman, 2023; Zamanifard et al., 2024).

Traditional medicine has historically valued polyherbal formulations that blend various botanical drugs to improve therapeutic benefits while reducing side effects. One such formulation, Ya-Samarn-Phlae, meaning “wound healing medicine” in Thai, has been historically used by folk healers in Southern Thailand to treat infected and chronic wounds (Chusri et al., 2012a). Ya-Samarn-Phlae comprises equal parts of dried or fresh pericarp from *Garcinia mangostana*, dried seeds from *Oryza sativa*, dried or fresh rhizomes from *C. longa*, and dried seeds from *Areca catechu*. The ethanol extract of Ya-Samarn-Phlae exhibits multiple *in vitro* biological activities relevant to wound healing, including antibacterial, anti-biofilm, antioxidant, anti-inflammatory, and fibroblast-stimulating effects (Chusri et al., 2013a; Chusri et al., 2013b; Chusri et al., 2012b).

Recent studies have demonstrated the clinical potential of an infused oil preparation derived from Ya-Samarn-Phlae (T-YaSP) in enhancing wound healing among diabetic patients and eliminating the biofilm of skin-related pathogens (Chokpaisarn et al., 2019; Sanpinit et al., 2024; Sanpinit et al., 2020). However, limitations such as an incomplete understanding of its mechanisms of action and practical challenges associated with its oily texture have been noted. To address these gaps, a novel gauze dressing impregnated with T-YaSP (YaSP) has been developed to overcome the challenges of its texture while maintaining its therapeutic efficacy. This present study additionally explores the wound-healing mechanisms of YaSP using a carrageenan-induced paw edema rat model and circular full-thickness skin wounds in Wistar and Goto-Kakizaki (GK) rats, which exhibit diabetic pathophysiology. This study aims to elucidate the biological mechanisms underlying the effects of YaSP and establish its potential as an alternative treatment for diabetic wounds.

## 2 Materials and methods

### 2.1 Plant materials, preparation of the traditional YaSP solution

Ya-Samarn-Phlae was prepared using equal quantities (100 g each) of dried and finely ground *C. longa* L. (Zingiberaceae; MTM04-ARDA003) rhizome, *A. catechu* L. (Palmae; MTM04-ARDA004) seed, *O. sativa* L. (Gramineae; MTM04-ARDA002) seed, and *G. mangostana* L. (Guttiferae; MTM04-ARDA001) pericarp. All botanical drugs were authenticated by Dr. Katesarin Maneenoon, and the voucher specimens were deposited at the Faculty of Traditional Thai Medicine, Prince of Songkla University, Hat Yai, Songkhla, Thailand, as previously published. These materials were sourced from reliable suppliers, ensuring consistency with previously validated research (Supplementary Table S1). The traditional YaSP solution was prepared according to a standardized procedure for infused oil, as detailed in Chokpaisarn et al. (2019) and Sanpinit et al. (2020).

### 2.2 Development of a novel dressing impregnated with the YaSP solution (YaSP)

A novel gauze dressing was developed by impregnating the YaSP-infused oil into an ointment base. Various formulations (Supplementary Table S2) were prepared using different ratios of white soft paraffin and hard paraffin to optimize the consistency and stability of the ointment. The mixtures were stirred in a water bath maintained at 60°C and evaluated for viscosity and homogeneity using a Brookfield viscometer (Model RVT; Spindle T-F meter). Adsorbent gauze (10 × 10 cm) was immersed in 50 mL of the optimized YaSP solution at 60°C for 10 s, drained for 40 s, and packaged in aluminum containers. The final product was stored at 25°C for subsequent experiments.

### 2.3 Stability testing of YaSP

The physical and chemical stability of YaSP was assessed under freeze-thaw conditions and long-term storage. Freeze-thaw testing involved five cycles of freezing at 4 °C for 24 h and thawing at 40°C for 24 h, with subsequent evaluation of viscosity, appearance, and concentrations of curcumin, α-mangostin, and MDA (Dantas et al., 2016). Long-term stability was evaluated at 25°C ± 2°C and 60% ± 5% relative humidity over 6 months in accordance with ICH Quality Guideline (2003). Subsequently, the chemical profiles and concentrations of specific chemical markers, including α-mangostin, curcumin, and arecoline, were quantitatively analyzed using LC-MS/MS and HPLC techniques, respectively (Chokpaisarn et al., 2019; Sanpinit et al., 2024; Sanpinit et al., 2020).

### 2.4 Skin irritation testing in rabbits

The impregnated dressing was evaluated for acute dermal irritation per OECD guideline 404 under the auspices of the Thailand Institute of Scientific and Technology Research (TISTR). Acute dermal irritation testing was conducted on three healthy New Zealand albino rabbits following OECD guideline 404 (OECD, 2014). A 10 × 10 cm section of dorsal fur was shaved, and 0.5 g of YaSP ointment was applied to a 2.5 × 2.5 cm gauze patch. The patch was secured with a transparent film dressing for 4 hours, after which the area was rinsed and observed for erythema, inflammation, or other adverse effects over 72 h. Observations were scored according to the primary dermal irritation index (PDII).

### 2.5 Anti-inflammatory effects of YaSP in carrageenan-induced paw edema rat model

The anti-inflammatory properties of YaSP were evaluated in a carrageenan-induced paw edema model using 60 male Wistar albino rats (150–200 g, 8 weeks old). All animal experiments were conducted following the ethical guidelines outlined by the Institutional Animal Ethics Committee (IAEC) at Jiangsu University, Zhenjiang, Jiangsu, China (approval number: SYXK-(SU)-20130036). Rats were housed under controlled conditions with *ad libitum* access to food and water, and their welfare was monitored throughout the study (Hussein et al., 2012).

Sample size determination was performed with G*Power software (Faul et al., 2007; Faul et al., 2009). A two-tailed independent t-test was chosen based on the *a priori* power analysis. Assumptions included a significance level (α) of 0.05, a statistical power (1–β) of 0.95, and an effect size of 1.5 derived from past research (Hussein et al., 2012). The minimum sample size calculated was 13 rats per group, but 15 rats were included to mitigate potential attrition and variability.

Animals were randomly allocated into three treatment groups (n = 15/group): Group A (negative control group) topically received 0.2 g of ointment without YaSP; Group B (positive control group) received 0.2 g of 5% (w/w) phenylbutazone, a non-steroidal anti-inflammatory drug; and Group C received 0.2 g of 25% (w/w) YaSP ointment.

Edema was induced by injecting 100 µL of 1% (w/v) carrageenan into the right hind paw of each rat. Paw thickness was measured at baseline and 1, 2, and 3 h post-injection using a digital vernier caliper. Rats were euthanized 3 hours post-injection, and paw tissue samples were collected for biochemical analysis of malondialdehyde (MDA), nitric oxide (NO), and myeloperoxidase (MPO) levels. ELISA kits were used to quantify inflammatory mediators, including TNF-α, IL-1β, and PGE_2_, as well as inducible nitric oxide synthase (iNOS) and cyclooxygenase-2 (COX-2) levels. Histological analysis was performed on hematoxylin and eosin-stained tissue sections to assess neutrophil infiltration. These sections were examined and photographed using a light microscope (Olympus Inc., Tokyo, Japan) to identify structural abnormalities.

### 2.6 Wound healing properties of YaSP in non-diabetic and type 2 diabetic wounds

The efficacy of wound healing was assessed in male Wistar albino and Goto-Kakizaki (GK) rats (180–200 g, 8 weeks old), which represent non-diabetic and type 2 diabetic models, respectively. The sample size was calculated using G*Power software (version 3.1.9.7, Heinrich-Heine-Universität Düsseldorf, Germany) (Faul et al., 2007; Faul et al., 2009). A two-tailed independent t-test was selected based on *a priori* power analysis. The assumptions included a significance level (α) of 0.05, a statistical power (1–β) of 0.95, and an effect size of 1.725 from previous research (Alsarayreh et al., 2022). The minimum sample size was 10 rats per group; however, 12 rats were used to account for potential attrition and variability.

Full-thickness wounds (6 mm diameter) were created on the dorsal skin under anesthesia (Kong et al., 2013). All experimental protocols were approved by the Institutional Animal Ethics Committee (IAEC) at Jiangsu University, Zhenjiang, Jiangsu, China (approval number: SYXK-(SU)-20130036), ensuring compliance with ethical standards for animal research. Rats were housed under standard laboratory conditions with controlled temperature (25°C ± 2°C), relative humidity (50%–60%), a 12-h light-dark cycle, and free access to food and water.

In a parallel experimental design, Wistar and GK rats were randomized into three groups (n = 18 Wistar rats and n = 12 GK rats per group): (1) the vehicle control group, (2) the Solcoseryl^®^ ointment group (MENARINI, Thailand), which served as the standard treatment and positive control, and (3) the YaSP ointment group, which was designated as the test group.

Treatments were applied every other day for 11 days. Wound areas were measured using digital calipers on days 0, 3, 5, 7, 9, and 11. Tissue samples were collected on days 7 (Wistar rats) and 11 (both Wistar and GK rats) to analyze transforming growth factor-β1 (TGF-β1), VEGF, collagen type I, and collagen type III levels using ELISA kits. Histological analysis (H&E and Masson’s trichrome staining) was performed to evaluate epithelialization, collagen deposition, and granulation tissue formation.

### 2.7 Statistic

All results are expressed as mean ± SEM or mean ± SD. Comparisons between the experimental and vehicle groups were conducted using one-way analysis of variance (ANOVA) with SPSS software Version 19.0 (IBM SPSS, Inc., Chicago, IL, http://www.ibm.com). A p-value of ≤0.05 was considered statistically significant.

## 3 Results

### 3.1 Stability and safety assessment of YaSP

The physical and chemical stability of YaSP under freeze-thaw cycles and long-term storage conditions demonstrated overall resilience with minimal changes. While the appearance of the dressing remained unchanged, minor alterations were noted in viscosity and concentrations of active ingredients, including curcumin and α-mangostin (Supplementary Figure S1; Supplementary Table S3). However, a significant increase in malondialdehyde (MDA) levels indicated mild product oxidation under these conditions. A qualitative analysis of the chemical profile showed changes during long-term storage for 6 months, except for curcumin, α-mangostin, demethoxycurcumin, dihydro rotenone, and methyl tanshinonate (Supplementary Table S4). Long-term storage over 6 months revealed a notable reduction in curcumin concentration, whereas α-mangostin levels remained stable (Table 1). These results highlight the need for optimized storage conditions to preserve the antioxidative and therapeutic properties of YaSP. Furthermore, the primary dermal irritation index (PDII) of YaSP was calculated as 0.17, classifying the ointment as non-irritating to slightly irritating. No significant erythema, inflammation, or other adverse skin reactions were observed during the 72-h evaluation period, supporting its safety for topical application (Supplementary Table S5).

**TABLE 1:** Effect of long-term storage on contents of metabolites, α-mangostin, curcumin, and arecoline and *in vitro* biological properties expressed as antioxidant and antiinflammation activities found in the ointment base for Ya-Samarn-Phlae tulle-gras dressings (YaSP).

Parameters	Contents (mg/g; mean ± SD) or percentage of inhibition (mean ± SD		
	Storage time (months)		
	Initial	3	6
*Metabolites (mg/g)*			
α-mangostin	0.42 ± 0.00	0.49 ± 0.00*	0.46 ± 0.00*
Curcumin	0.37 ± 0.00	0.31 ± 0.00*	0.18 ± 0.00*
Arecoline	<LOD	<LOD	<LOD
*Biological activities (% of inhibition)*			
Nitric oxide scavenging	41.14 ± 0.94	37.06 ± 0.82*	26.43 ± 0.94*
Lipid peroxidation inhibition	14.59 ± 1.48	11.39 ± 1.88*	10.44 ± 0.97*

*p < 0.05, significantly different from the initial. (Independent samples T-Test); Limit of detection (LOD) arecoline = 0.001 mg/g. Catechin (0.63 mg/mL) and BHT (10% v/v) were included as positive controls for nitric oxide (NO) scavenging activity and lipid peroxidation inhibition expressed percentage of inhibition of 52.95 ± 3.19 and 89.06 ± 1.00, respectively.

### 3.2 YaSP mitigated the carrageenan-induced paw inflammatory response by reducing the production of inflammatory cytokines and mediators and diminishing oxidative stress

**FIGURE 1:**
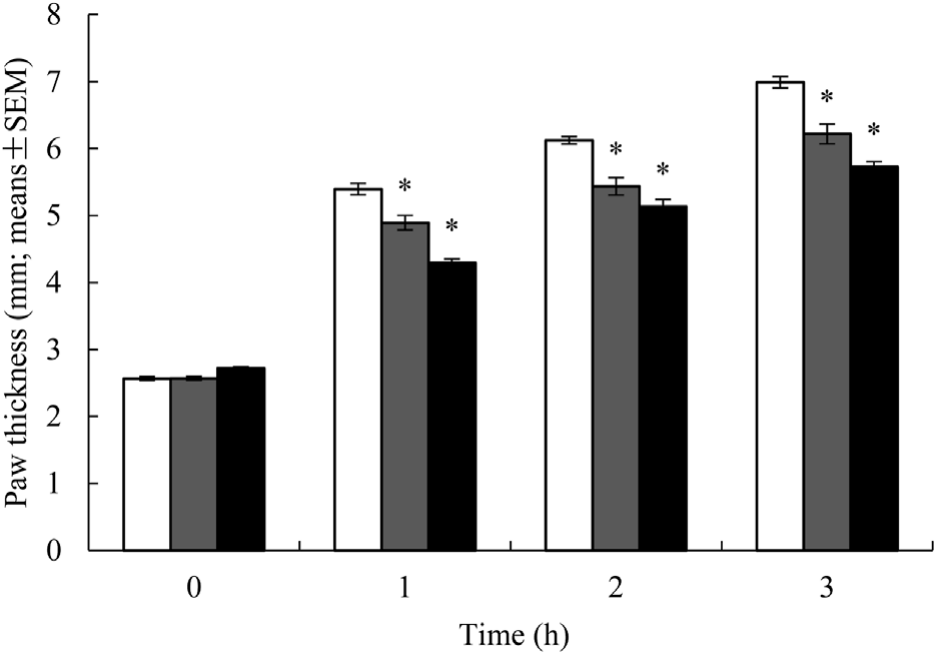
Paw thickness in rats treated with ointment base for Ya-Samarn-Phlae tulle-gras dressings (YaSP; black bars), 5% (w/v) of phenylbutazone (grey bars), and vehicle-treated group (white bars) on carrageenan-induced paw edema model. Paw thickness degree was presented as the mean ± SEM of 15 rats per group, *p < 0.001 vs. vehicle.

The well-established carrageenan-induced rat paw edema model was utilized to evaluate the potential anti-inflammatory effects of YaSP. This product treatment significantly reduced paw thickness compared to the vehicle group (p < 0.001), with results comparable to the positive control (phenylbutazone). The reduction in edema was observed in a time-dependent manner, indicating the efficacy of YaSP in mitigating inflammation (Figure 1).

**FIGURE 2:**
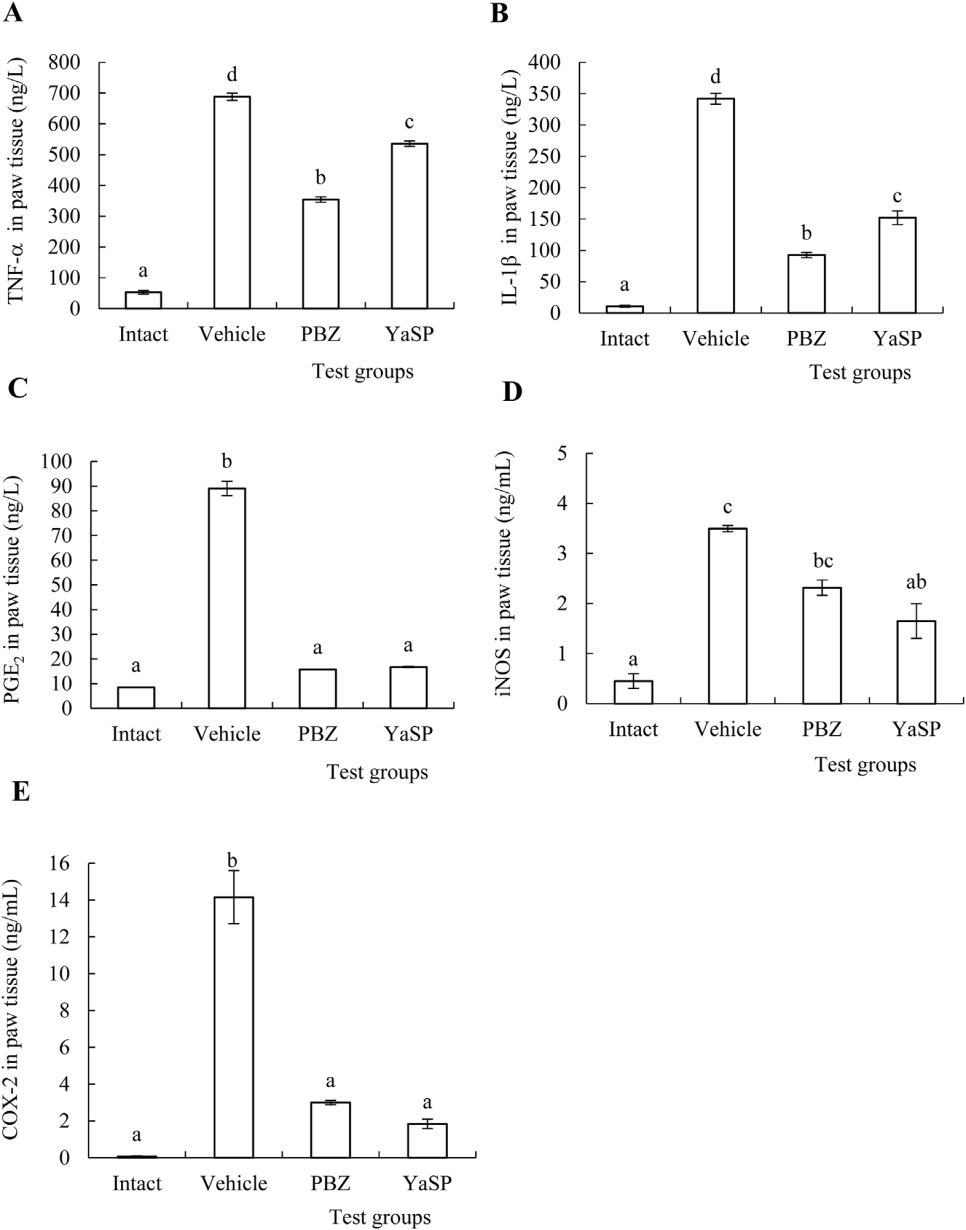
Effect of ointment base for Ya-Samarn-Phlae tulle-gras dressings (YaSP) on the concentrations of inflammatory cytokines, TNF-α **(A)**, IL-1β **(B)** and PGE_2_ **(C)** and pro-inflammatory enzymes, iNOS **(D)** and COX-2 **(E)** in the rat paws treated with carrageenan. The values represent the mean ± SEM of five rats. ^(a–d)^ Values in the same factor followed by a different letter are significantly different (*p* < 0.001). Phenylbutazone (PBZ, 5%; w/w) was used as a positive control.

The levels of malondialdehyde (MDA), nitric oxide (NO), and myeloperoxidase (MPO) in paw tissue were significantly elevated in the vehicle group following carrageenan injection. Treatment with YaSP significantly reduced these markers of oxidative stress and inflammation (p < 0.01), matching the efficacy of phenylbutazone (Table 2). Additionally, YaSP demonstrated a pronounced effect in lowering inflammatory cytokines and mediators, including TNF-α, IL-1β, and PGE_2_, as well as the pro-inflammatory enzymes inducible nitric oxide synthase (iNOS) and cyclooxygenase-2 (COX-2) (Figure 2).

**TABLE 2:** Effect of the ointment base for Ya-Samarn-Phlae tulle-gras dressings (YaSP) on the production of malondialdehyde (MDA), nitric oxide (NO), and myeloperoxidase (MPO) in carrageenan-induced paw edema in rats.

Test groups	malondialdehyde	nitric oxide	myeloperoxidase
	(nmol/mg protein)	(µmol/g protein)	(U/g tissue)
intact	1.70 ± 0.03^a^	22.56 ± 1.19^a^	0.07 ± 0.01^a^
vehicle	6.05 ± 0.16^c^	118.66 ± 2.06^c^	0.33 ± 0.01^c^
phenylbutazone 5%; (w/w)	3.36 ± 0.12^b^	39.63 ± 0.81^b^	0.25 ± 0.01^b^
YaSP	3.37 ± 0.16^b^	32.10 ± 1.03^b^	0.21 ± 0.01^b^

Each value represents the mean ± SEM, of five rats per group. ^(a–c)^ Values in the same column followed by a different letter are significantly different (*p* < 0.001). (One-way ANOVA, followed by Duncan).

**FIGURE 3:**
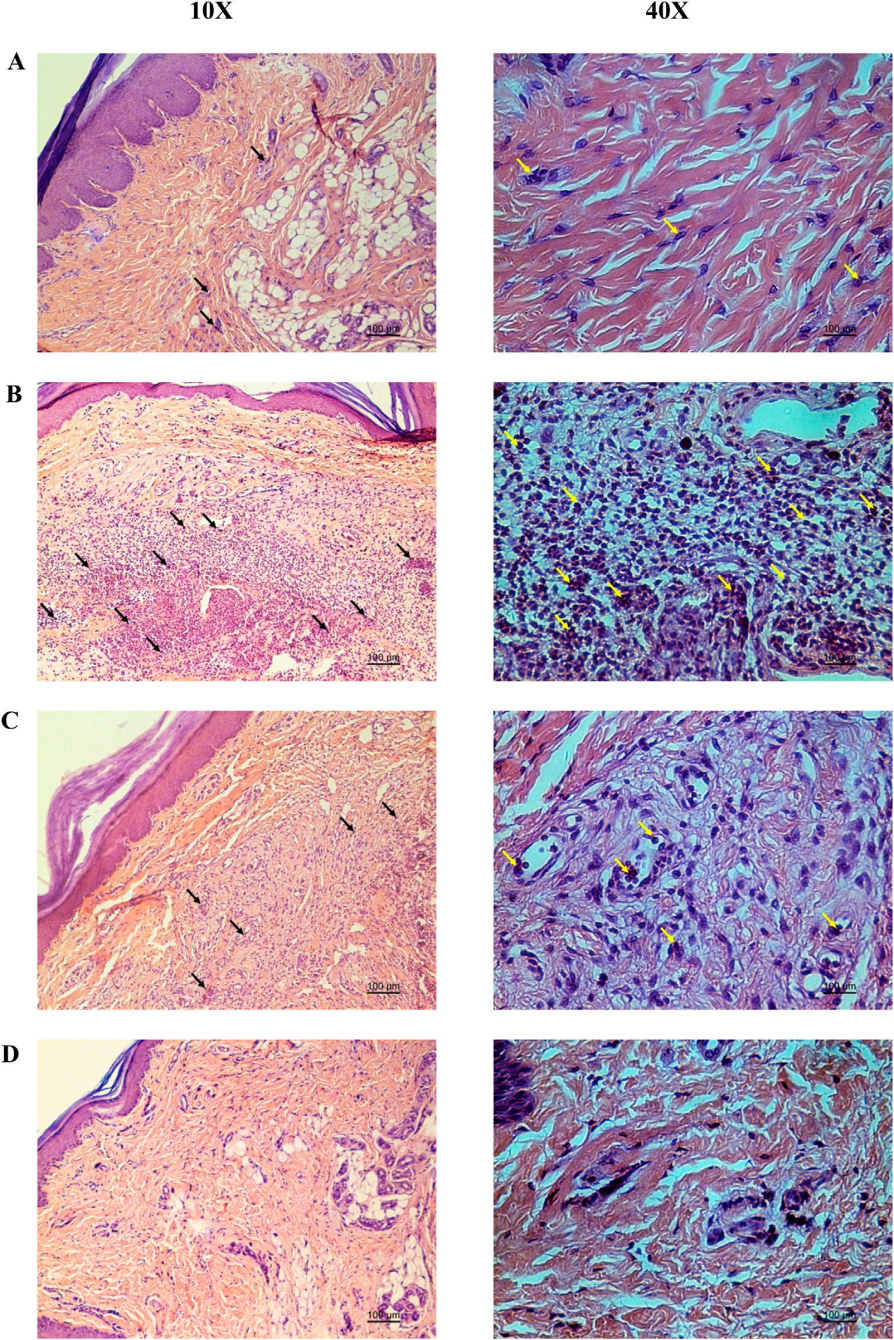
Representative photomicrographs from the histopathological analysis of rat paw tissue demonstrate the protective effect of the Ya-Samarn-Phlae ointment base used with tulle-gras dressings (YaSP; **(A)** in comparison to the carrageenan-induced rat paw edema treated with a vehicle **(B)** or the positive control, phenylbutazone [**(C)**, 5%; w/v], along with the intact group **(D)**. Black and yellow arrows indicate neutrophils. Scale bar: 100 µm.

**FIGURE 4:**
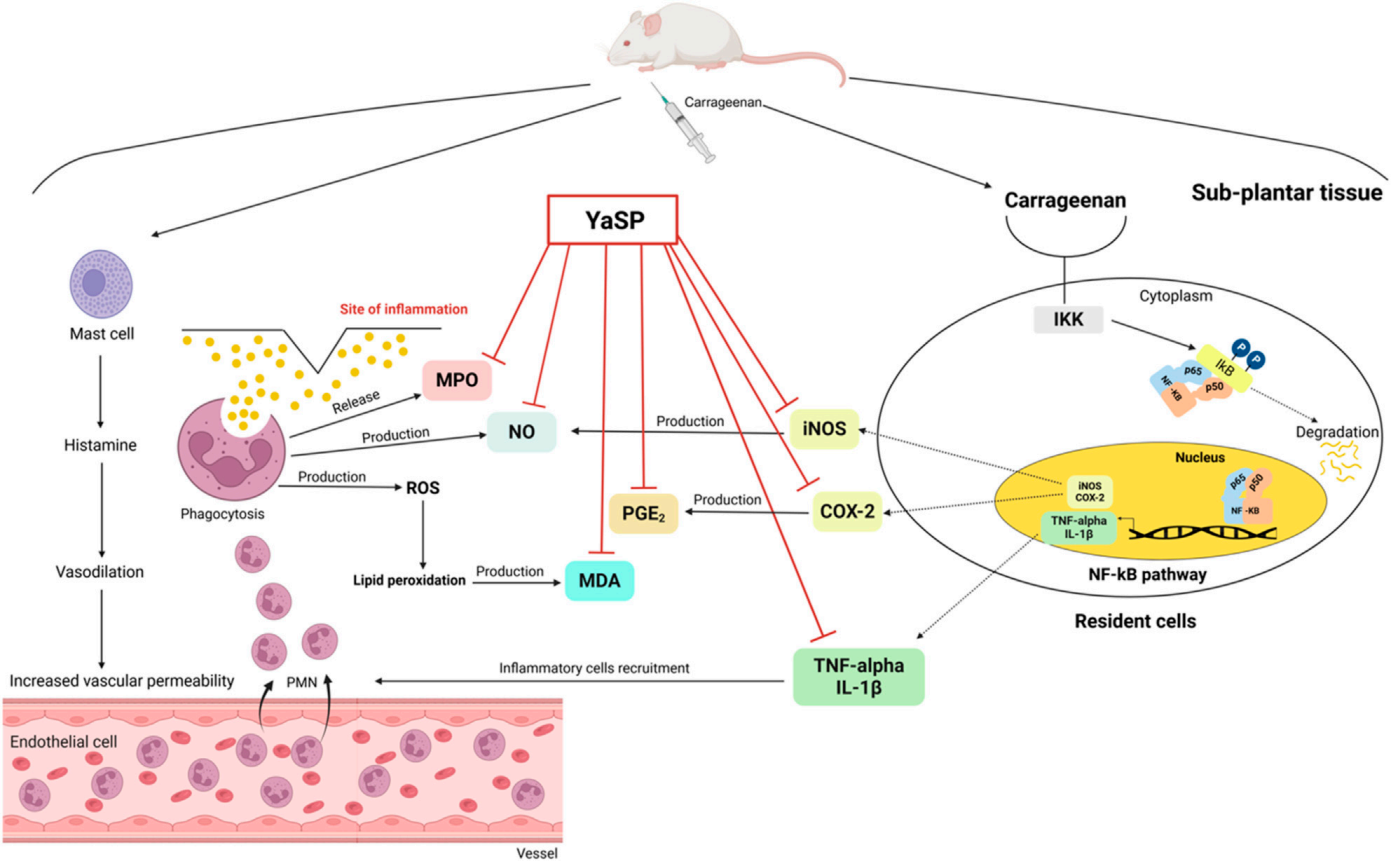
Schematic illustration of the proposed anti-inflammatory mechanism of the ointment base for Ya-Samarn-Phlae tulle-gras dressings (YaSP) in carrageenan-induced paw edema in rats. Carrageenan induces inflammation by activating the NF-κB signaling pathway, which triggers the production of inflammatory mediators (TNF-α, IL-1β, iNOS, COX-2). Additionally, activated mast cells release histamine, leading to vascular changes and leukocyte recruitment. Leukocytes contribute to inflammation by releasing MPO, nitric oxide, and reactive oxygen species, resulting in lipid peroxidation (increased MDA) and prostaglandin E2 (PGE_2_) production. Treatment with YaSP effectively suppresses inflammation by inhibiting critical inflammatory enzymes and mediators (MPO, NO, MDA, PGE_2_, iNOS, COX-2, TNF-α, IL-1β).

Hematoxylin and eosin-stained sections showed marked reductions in neutrophil infiltration and tissue swelling in the YaSP-treated group compared to the vehicle group (Figure 3). These findings confirm the anti-inflammatory potential of YaSP. In summary, Figure 4 illustrates that YaSP exhibits substantial anti-inflammatory effects comparable to phenylbutazone. This is demonstrated by its capacity to diminish the levels of critical inflammatory cytokines and enzymes in a carrageenan-induced inflammation model in rats. Its mechanism of action involves the modulation of iNOS and COX-2 activation, which subsequently influences the levels of the end-product inflammatory mediators NO and PGE_2_. Additionally, treatment with YaSP significantly reduces the levels of MPO, TNF-α, IL-1β, and MDA, which are also significant contributors to the inflammatory phase of wound healing.

### 3.3 Topical application of YaSP significantly accelerates the healing of non-diabetic wounds by enhancing their proliferation and remodeling phases, as indicated by the changing levels of TGF-β1, VEGF, collagen I, and collagen III

**FIGURE 5:**
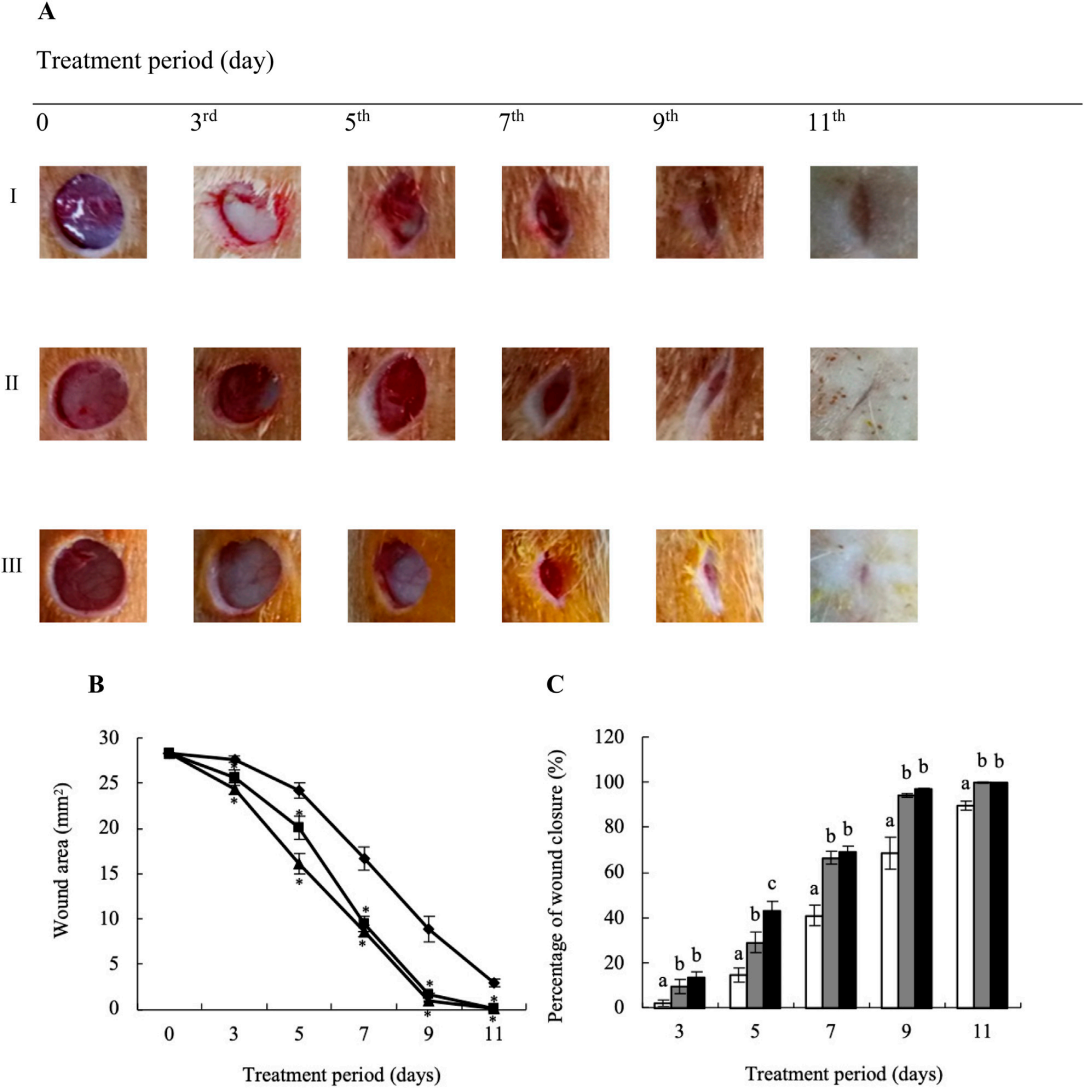
Representative macrophotographs of wounds **(A)**, wound areas **(B)**, and percentage of wound closure **(C)** of non-diabetic wounds in Wistar rats, measured on the operation day (day 0) and on days 3, 5, 7, 9 and 11 after daily treatment with vehicle (I), Solcoseryl^®^ ointment (a positive control; II), or the ointment base for Ya-Samarn-Phlae tulle-gras dressings (YaSP; III). The results represent the mean ± SEM of 9–10 animals per group. *p < 0.05 vs. the control. ^(a–c)^ Values on the same days followed by a different letter differ significantly (*p* < 0.05).

YaSP significantly enhanced wound closure rates in non-diabetic Wistar rats. By day 7, YaSP-treated wounds exhibited a reduction in wound area of 64% compared to the vehicle group, achieving over 90% closure by day 11. The average wound area in the vehicle-treated group diminished from 27.63 ± 0.36 mm^2^ on day 3–16.66 ± 1.27 mm^2^ on day 7 and to 2.91 ± 0.39 mm^2^ by day 11. In comparison, the group treated with YaSP exhibited a more pronounced decrease in wound area from 24.37 ± 0.63 mm^2^ on day 3–8.66 ± 0.62 mm^2^ on day 7, culminating in a minimal 0.10 ± 0.04 mm^2^ by day 11. These results were found to be in line with those of the positive control, where the average wound area shrank from 25.56 ± 0.85 mm^2^ on day 3–9.45 ± 0.86 mm^2^ on day 7, and further to 0.07 ± 0.03 mm^2^ by day 11. These results were comparable to those observed in the positive control group treated with Solcoseryl^®^ ointment (Figure 5).

**FIGURE 6:**
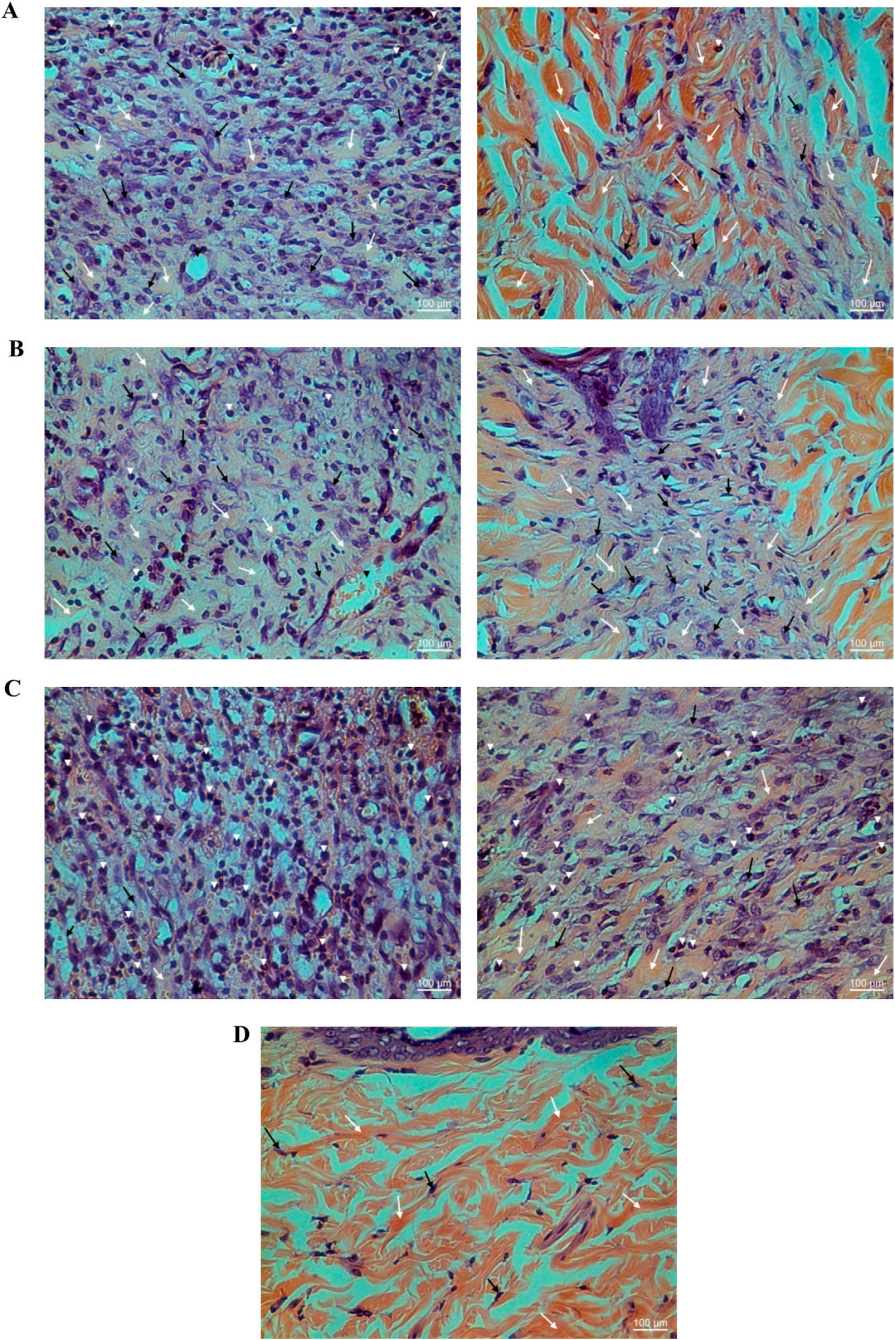
Histological examination of hematoxylin-eosin (HE) stained non-diabetic wound tissues treated with the ointment base for Ya-Samarn-Phlae tulle-gras dressings (YaSP; **(A)**, Solcoseryl^®^ ointment [reference product; **(B)**], vehicle **(C)**, along with normal tissue **(D)** on the seventh (left panel) and 11th days (right panel). The white arrows, black arrows, and arrowheads indicate the amounts of collagen, fibroblasts, and small numbers of inflammatory cells, respectively. (Magnification ×40; scale bars: 100 µm).

**FIGURE 7:**
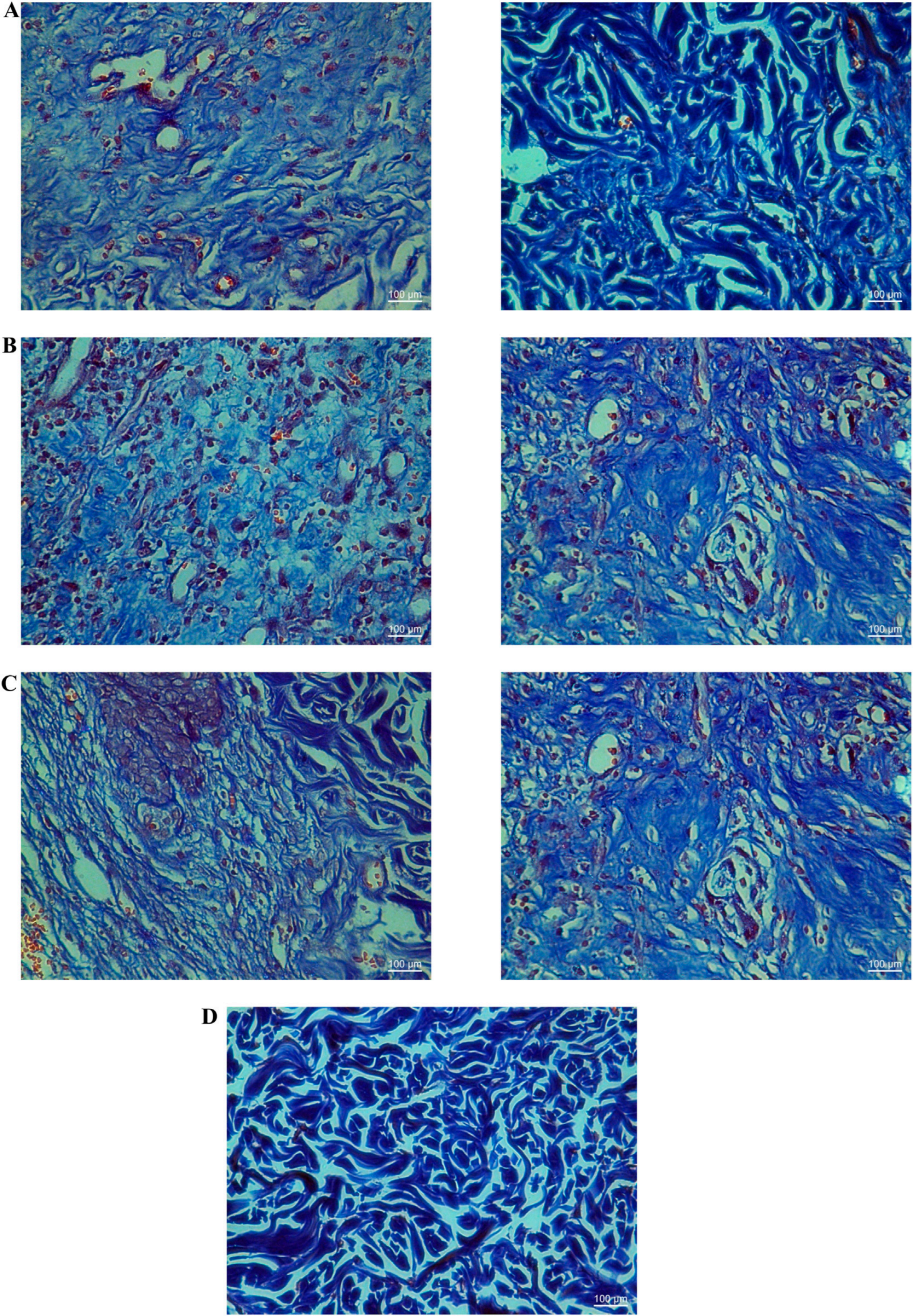
Histological examination of Masson’s trichrome stained tissues from non-diabetic wounds treated with the ointment base for Ya-Samarn-Phlae tulle-gras dressings (YaSP; **(A)**, a vehicle **(B)**, and Solcoseryl^®^ ointment (a reference product; **(C)**, along with normal tissue **(D)** on the seventh (left panel) and 11th days (right panel). The dark blue area indicates the amount of collagen. (Magnification ×40; scale bars: 100 µm).

YaSP treatment significantly increased levels of collagen type III during the proliferation phase (day 7), followed by a substantial rise in collagen type I during the remodeling phase (day 11) (Table 3). These changes were validated by Masson’s trichrome staining, which showed greater collagen density and thickness in YaSP-treated wounds compared to controls (Figure 6). VEGF and TGF-β1 levels were significantly elevated in YaSP-treated wounds, indicating enhanced angiogenesis and granulation tissue formation (Table 3). Histological sections confirmed increased fibroblast activity and neovascularization in the YaSP group compared to the vehicle group (Figure 7).

**TABLE 3:** The levels of transforming growth factor β 1 (TGF-β1), vascular endothelial growth factor (VEGF), collagen type I, and collagen type III in non-diabetic wounds after being treated with ointment base for Ya-Samarn-Phlae tulle-gras dressings (YaSP).

Parameters	Treatment	Test groups		
	Period (days)	Vehicle	Solcoseryl^®^ ointment	YaSP
TGF-β1 (ng/L)	0	73.74 ± 1.21		
	7	147.50 ± 1.17*^,a^	259.16 ± 12.38*^,c^	201.72 ± 5.48*^,b^
	11	131.87 ± 0.70*^,c^	111.51 ± 0.43*^,a^	118.70 ± 0.51*^,b^
VEGF (ng/L)	0	60.20 ± 3.51		
	7	182.08 ± 1.03*^,a^	330.89 ± 7.20*^,b^	356.47 ± 6.38*^,b^
	11	241.74 ± 2.78*^,b^	212.95 ± 5.07*^,a^	206.76 ± 3.98*^,a^
Collagen I (ng/mL)	0	134.76 ± 3.54		
	7	47.19 ± 0.76*^,a^	103.09 ± 4.69*^,c^	83.82 ± 3.99*^,b^
	11	80.68 ± 4.30*^,a^	124.26 ± 2.35*^,b, c^	115.70 ± 1.49*^,b^
Collagen III (ng/mL)	0	15.71 ± 1.42		
	7	36.88 ± 0.59*^,a^	124.26 ± 2.35*^,b, c^	74.86 ± 4.24*^,b^
	11	52.07 ± 3.75*^,b^	28.25 ± 0.94*^,a^	29.78 ± 3.26*^,a^

The results represent the mean ± SEM, of 4-5 animals per group. *p < 0.001 vs. the day 0. ^(a–c)^ Values on the same days followed by a different letter are significantly different (*p* < 0.001). Solcoseryl^®^ ointment was used as a reference product.

### 3.4 Promoting granulation tissue formation, angiogenesis, and collagen synthesis following treatment with YaSP significantly accelerates wound healing in the type 2 diabetes model

The well-established Goto-Kakizaki (GK) rat model was utilized to explore further the potential of YaSP topical treatment to enhance the wound healing rate in type 2 diabetics. This nonobese, nonhypertensive type 2 diabetes model simulates the development of peripheral neuropathy and delayed epithelial regeneration, which in turn slows down wound healing, a condition also observed in diabetic patients. The wound healing experiments noted no significant difference in blood glucose concentrations among GK rats in each group, ranging from 10.28 to 12.98 mmol/L (Supplementary Figure S2).

**FIGURE 8:**
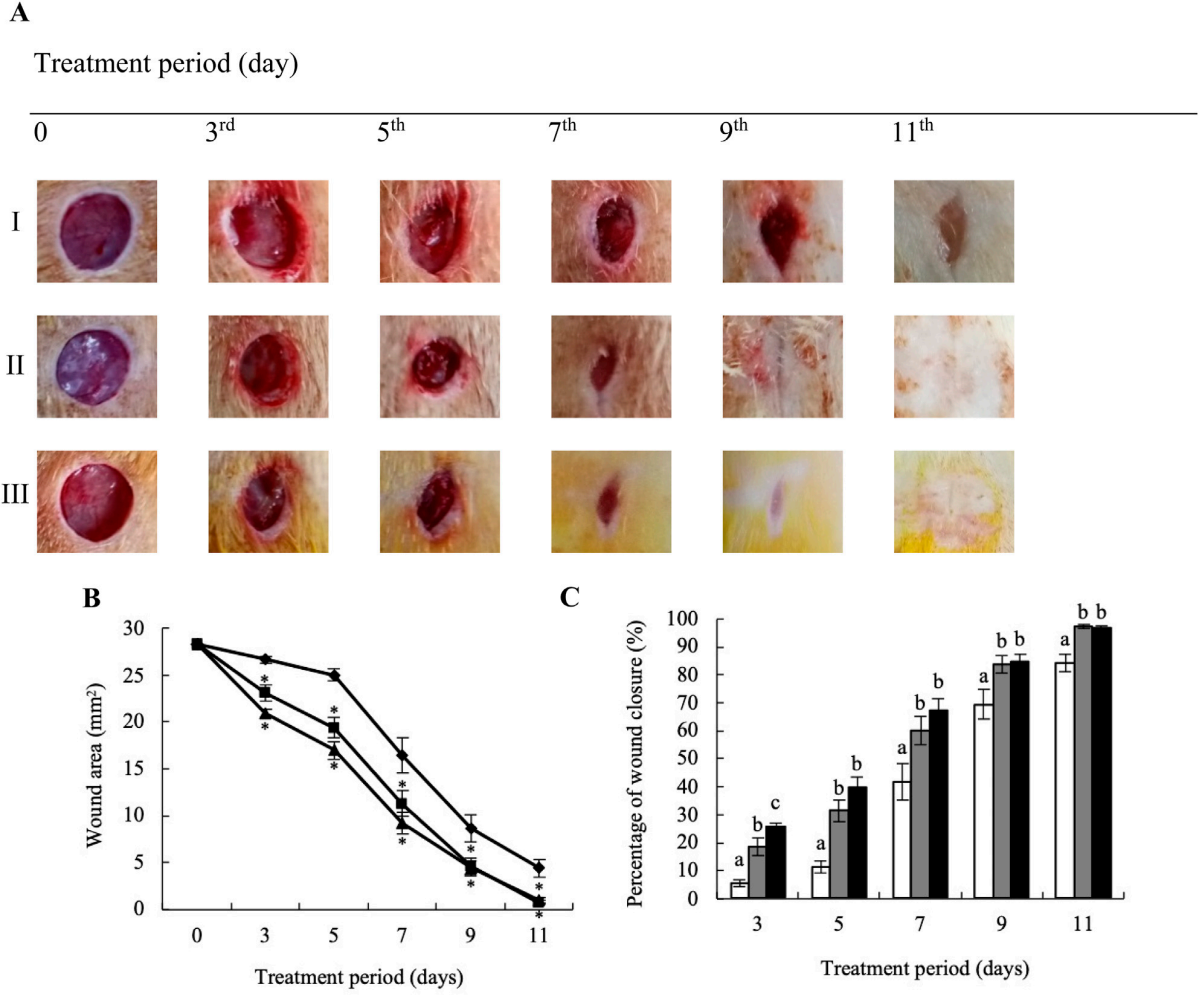
Representative macrophotographs of wounds **(A)**, wound areas **(B)**, and percentage of wound closure **(C)** in type 2 diabetic Goto-Kakizaki rats were measured on the day of the operation (day 0) and on days 3, 5, 7, 9, and 11 following daily treatment with vehicle (I), Solcoseryl^®^ ointment (a reference product; II), or the ointment base for Ya-Samarn-Phlae tulle-gras dressings (YaSP; III). The results represent the mean ± SEM of 9–10 animals per group. *p < 0.05 compared to the control. (a–c) Values on the same days followed by a different letter are significantly different at p < 0.05.

**FIGURE 9:**
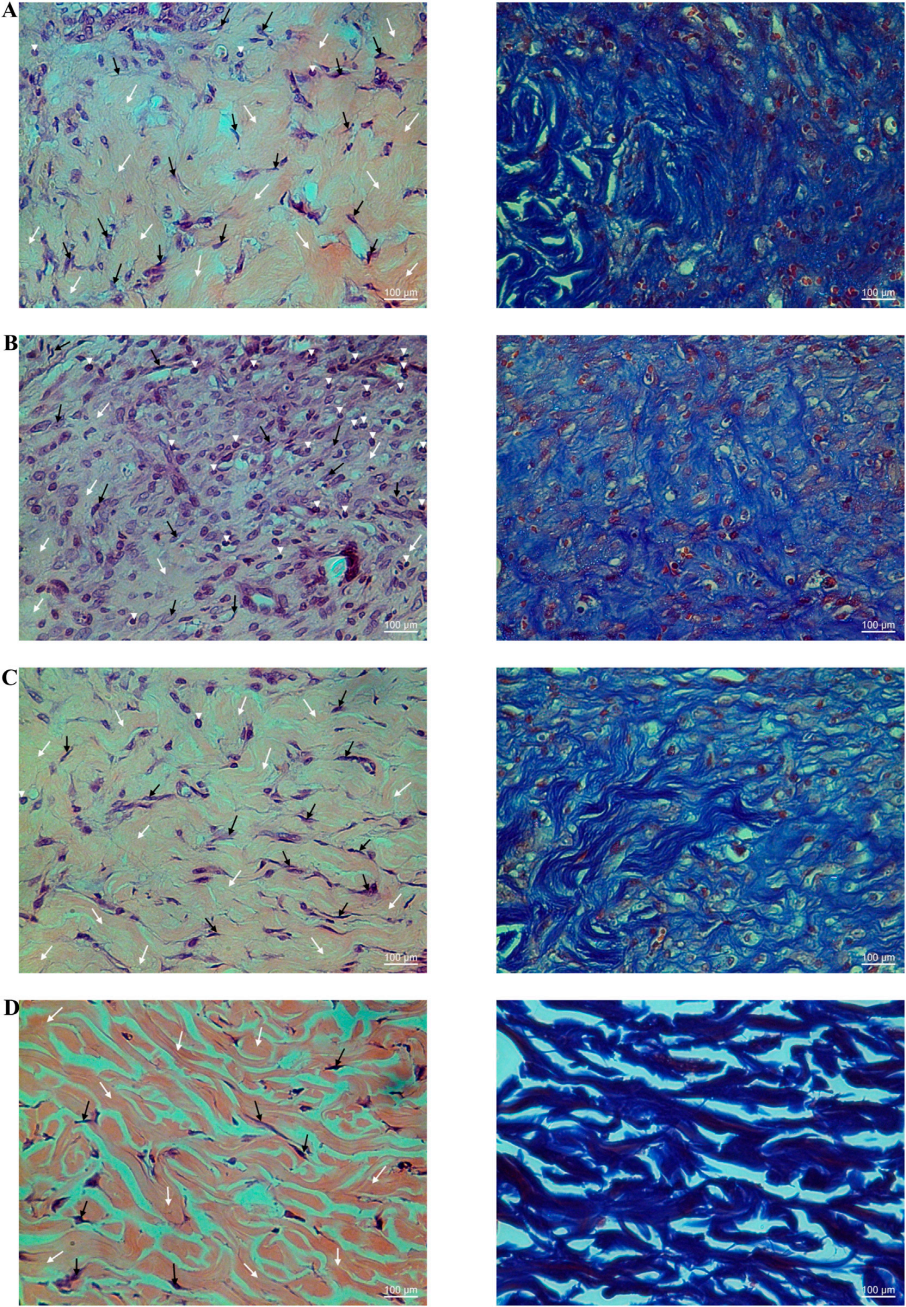
Histological examination of hematoxylin-eosin (H&E; left panel) and Masson’s trichrome (right panel) stained diabetic wound tissues treated with the ointment base for Ya-Samarn-Phlae tulle-gras dressings (YaSP; **(A)**, a vehicle **(B)**, Solcoseryl^®^ ointment (a reference product; **(C)**, along with normal tissue **(D)** on the 11th day. In the left panels, the white and black arrows and arrowheads indicate the amounts of collagen, fibroblasts, and a small number of inflammatory cells. In the right panels, the dark blue area represents the amount of collagen (Magnification ×40; scale bars: 100 µm).

Goto-Kakizaki (GK) rats treated with YaSP exhibited significantly improved wound closure compared to the vehicle group. By day 11, YaSP-treated wounds achieved 96.8% closure, closely matching the positive control (97.4%), while the vehicle-treated group showed only 84.4% closure (Figure 8). YaSP treatment significantly increased TGF-β1 and VEGF levels and the ratio of collagen type III to type I in diabetic wounds. This highlights its role in promoting angiogenesis during the proliferation phase and facilitating collagen remodeling during the later stages of healing (Table 4). Histological analysis showed enhanced epithelialization and reduced inflammatory cell infiltration in YaSP-treated wounds (Figure 9). The elevated levels of TGF-β1 and VEGF in YaSP-treated wounds suggest a mechanistic basis for its efficacy in accelerating granulation tissue formation and angiogenesis. Reducing pro-inflammatory mediators further underscores its dual role in resolving inflammation and promoting tissue repair.

**TABLE 4:** The levels of transforming growth factor β 1 (TGF-β1), vascular endothelial growth factor (VEGF), collagen type I, and collagen type III in type 2 diabetic Goto-Kakizaki rats after treated with ointment base for Ya-Samarn-Phlae tulle-gras dressings (YaSP).

Parameters	Treatment	Test groups		
	Period (days)	Vehicle	Solcoseryl^®^ ointment	YaSP
TGF-β1	0	33.16 ± 0.81		
(ng/L)	11	49.18 ± 1.48*^,a^	114.06 ± 3.54*^,c^	99.90 ± 1.23*^,b^
VEGF	0	28.78 ± 0.12		
(ng/L)	11	97.20 ± 1.37*^,a^	188.71 ± 5.10*^,b^	201.81 ± 1.46*^,b^
Collagen I	0	103.95 ± 0.45		
(ng/mL)	11	52.64 ± 0.19*^,a^	100.47 ± 0.93*^,c^	97.15 ± 0.33*^,b^
Collagen III	0	20.27 ± 0.17		
(ng/mL)	11	65.80 ± 1.24*^,c^	22.05 ± 0.15^a^	24.99 ± 0.41*^,b^

The results represent the mean ± SEM, of 5-6 animals per group. *p < 0.001 vs. the day 0. ^(a–c)^ Values on the same days followed by a different letter are significantly different (*p* < 0.001).

**FIGURE 10:**
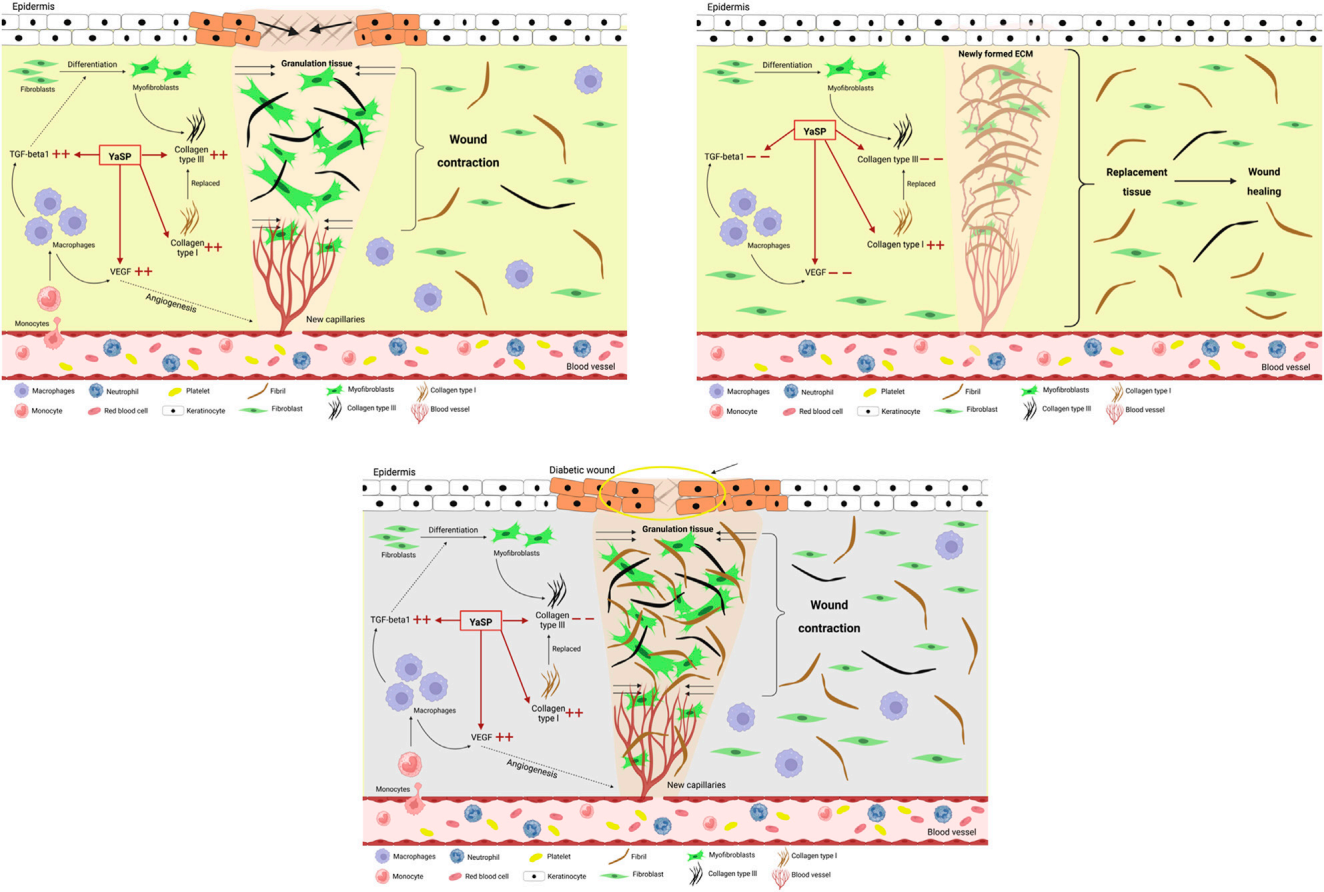
Schematic diagram of the proposed wound healing mechanisms of the ointment base for Ya-Samarn-Phlae tulle-gras dressings (YaSP) in non-diabetic Wistar rats and in type 2 diabetic Goto-Kakizaki rats. In non-diabetic wounds, YaSP reduces excessive TGF-β1 and VEGF expression, regulates fibroblast-to-myofibroblast differentiation, and balances collagen types I and III deposition, thereby promoting angiogenesis, ECM formation, wound contraction, and effective wound closure. In diabetic wounds, characterized by persistent inflammation, excessive collagen type I deposition, and abnormal fibroblast differentiation, YaSP reduces pathological levels of TGF-β1 and VEGF, normalizing angiogenesis, ECM remodeling, and fibroblast activity, thus significantly enhancing impaired diabetic wound healing.

In summary, as depicted in Figure 10, The results demonstrate that YaSP possesses significant anti-inflammatory and wound-healing properties in both non-diabetic and diabetic models. Its ability to reduce oxidative stress, enhance collagen synthesis, and stimulate angiogenesis positions it as a promising alternative for managing chronic wounds, particularly in diabetic patients. Future studies should focus on optimizing storage stability and further elucidating its molecular mechanisms of action.

## 4 Discussion

This present investigation was conducted in order to elucidate the mechanisms of action of an ointment formulated with a traditional Thai polyherbal preparation, T-YaSP, which is utilized as the active ingredient in a novel gauze dressing since previous reports have highlighted its remarkable wound-healing efficacy in diabetic patients (Sanpinit et al., 2024; Sanpinit et al., 2020). Our preliminary results conducted in type 2 diabetic GK rats reveal that YaSP could be a potent wound-healing agent (Sanpinit et al., 2018). The findings from this study demonstrate the underlying mechanisms of YaSP as a promising therapeutic agent for wound healing, particularly in diabetic contexts. YaSP exhibits potent anti-inflammatory, antioxidant, angiogenesis-enhancing, and tissue-regenerative properties, which have the potential to overcome critical challenges in diabetic wound healing, including chronic inflammation, impaired angiogenesis, and delayed epithelialization (Burgess et al., 2021; Patel et al., 2019; Worsley et al., 2023).

In diabetic foot ulcers, excess and delayed inflammation play critical roles in unhealing wounds (Worsley et al., 2023). The carrageenan-induced paw edema model highlighted significant anti-inflammatory effects of YaSP, as evidenced by reduced paw thickness and lower levels of key inflammatory biomarkers, including TNF-α, IL-1β, PGE_2_, iNOS, and COX-2, indicating that YaSP effectively modulates inflammatory pathways. Additionally, the observed reduction in oxidative stress markers such as MDA, MPO, and NO underscores its role in mitigating inflammation-associated oxidative damage. The inhibition of the activation of the nuclear factor kappa B (NF-κB) pathway that was well-established in α-mangostin (Pan et al., 2017) and curcumin (Li et al., 2019), the chemical markers of YaSP, was hypothesized as one of the mechanisms involving its anti-inflammatory and anti-oxidative stress properties observed in this study. This pathway is crucial in delaying diabetic wound healing, leading to prolonged inflammation, impaired angiogenesis, and decreased cell proliferation (Cai et al., 2023). In addition to its marker compounds, individual botanical drugs of YaSP, including *C. longa* (Lee et al., 2020), *G. mangostana* (Muniroh et al., 2021), *A. catechu* (Janpaijit et al., 2024), and *O. sativa* (Ha et al., 2016), have also been reported to attenuate the activation inhibition of the NF-κB pathway and subsequently reduce pro-inflammatory cytokines, such as TNF-α, IL-1β, and interleukin 6 (IL-6), and interleukin 8 (IL-8). In addition, curcumin (Zhao et al., 2015) and α-mangosteen (Chen et al., 2008) have been reported to inhibit iNOS and COX-2, which could consequently reduce NO and PGE_2_ production levels found in YaSP-treated tissue. These anti-inflammatory results indicate accelerated resolution of the inflammatory phase, which is critical for transitioning to subsequent wound healing stages. The ability of YaSP, which is at least partly mediated by its chemical markers, to balance pro- and anti-inflammatory mediators highlights its potential to overcome chronic inflammation, one of the important challenges in diabetic wound management.

In non-diabetic Wistar rats and the GK rat models of type 2 diabetes, YaSP significantly enhanced wound closure rates, achieving over 90% closure by day 11, comparable to the positive control. Enhanced VEGF and TGF-β1 levels indicate its mechanisms of action in promoting angiogenesis and granulation tissue formation, which is crucial for effective wound healing (Burgess et al., 2021; Patel et al., 2019). This rapid healing was additionally hypothesized to be associated with increased collagen synthesis and remodeling, evidenced by elevated levels of collagen type III during the proliferation phase and collagen type I during the remodeling phase. Both clinical and experimental studies have revealed that disrupted production of VEGF and TGF-β impairs wound healing in diabetic conditions (Al-Mulla et al., 2011; Altavilla et al., 2001; Jude et al., 2002; Krishnan et al., 2007), while scientific reports suggested that treatment with VEGF and TGF-β significantly improves wound healing in diabetic rats (El Gazaerly et al., 2013; Yan et al., 2010). Various *in vitro* experiments and animal models, including diabetic rats, have demonstrated that curcumin enhances the expression and production of VEGF and TGF-β (Chen et al., 2024; Kant et al., 2015), thereby confirming their role in promoting neovasculogenesis and accelerating wound healing. Limited information has been reported about α-mangostin, *G. mangostana*, arecoline, *A. catechu*, and *O. sativa,* indicating that these compounds may stimulate the expression and production of VEGF and TGF-β in human gingival fibroblasts, macrophages under glucose-induced conditions, dermal fibroblasts, and human hair follicle dermal papilla cells (Hsieh et al., 2018; Khantham et al., 2023; Lee et al., 2013; Patrick et al., 2024; Rizqiawan et al., 2021).

In addition to its influence on the NF-κB pathway, based on the obtained wound healing results, the HIF-1α/VEGF and the TGF-β/Smad signaling pathways could potentially be relevant to the diabetic wound healing mechanisms of YaSP and its metabolites. Chronic hyperglycemia can disrupt oxygen homeostasis, resulting in tissue hypoxia, leading to downregulating the hypoxia-inducible factor-1α (HIF-1α) (Catrina and Zheng, 2021). It has been well-established that the decrease in the transcriptional activity of HIF-1 leads to a downregulation of target genes essential for wound healing, including VEGF, which promotes the angiogenesis and causes the reduction of neovascularization resulting in delayed or nonhealing of chronic diabetic wounds (Catrina and Zheng, 2021; Zhu et al., 2024). Treating wounds of diabetic animal models and individuals with traditional polyherbal formulas, plant-derived metabolites, and several long non-coding RNAs have been shown to increase the expression of HIF-1α and VEGF in wounds and subsequently enhance the healing rates (Herman and Herman, 2023; Kumar et al., 2023; Tang et al., 2022; Zhou et al., 2022; Nusantoro et al., 2024b). The excess levels of TGF-β1 can promote angiogenesis by up-regulating VEGF during the early stage of wound healing, as shown in YaSP-treated wounds. However, it should be noted that elevated TGF-β1 levels can result in excessive fibroblast proliferation and scar formation during the later stages of wound healing (Campaner et al., 2006). Treatment of non-diabetic wounds with YaSP was found to increase TGF-β1 levels on the seventh day, followed by a reduction on the 11th day. This suggests that, in addition to promoting wound healing, YaSP may additionally reduce scar formation. This effect is reflected in the reduction of TGF-β1 levels, a decrease in type III collagen synthesis, and enhanced type I collagen synthesis during the later stages of wound healing. Based on accumulated knowledge, only a limited number of compounds and extracts have been identified to exhibit all these biological properties, such as triterpenoids from *C. asiatica* (Tang et al., 2011), epigallocatechin gallate (Sriram et al., 2015), moist exposed burn ointment (Gong et al., 2022; Jurjus et al., 2007), and enzymatically digested velvet antler peptides (Zhang et al., 2021).

Despite significant efforts and advancements in diabetic wound care by pharmaceutical industries, practical solutions remain limited, particularly for patients in LMICs. This study highlights the potential of the traditional Thai medicine, YaSP, for managing non-diabetic and diabetic wounds. YaSP has been demonstrated to possess antioxidant, antiinflammation, antibacterial, antibiofilm, and fibroblast growth-stimulating activities. Furthermore, it has been clinically validated as a promising therapeutic agent for diabetic ulcers. YaSP has shown the ability to reduce excessive inflammation, enhance angiogenesis, and regulate collagen metabolism during the remodeling phase of wound healing for the first time in type 2 diabetic and non-diabetic animal models.

The limitation concerning the translational relevance of the GK rat, a nonobese, nonhypertensive model of type 2 diabetes, to diabetic patients has been acknowledged as a restriction on the generalizability and translational relevance. Therefore, it is essential to examine the clinical implications of animal models for human diabetic wound healing, particularly in relation to other metabolic syndrome conditions, such as obesity and hypertension, to ensure effective implementation. The molecular mechanisms underlying the wound-healing properties of this formulation and its skin absorption properties are required for further investigation. YaSP for wound healing tends to be multiple-target focused due to the complex nature of its compounds. Therefore, utilizing the network pharmacology to predict and verify interactions between active ingredients and promising signaling pathways is suggested for further investigation.

## 5 Conclusion

In summary, our experiments provide comprehensive details on the mechanisms of action of YaSP for wound healing observed in both type 2 diabetic and non-diabetic animal models for the first time. YaSP was successfully developed, and its promising chemical markers, α-mangostin and curcumin, were monitored during long-term storage conditions. The wound-healing mechanisms of YaSP were hypothesized to be as follows: (i) reduce excessive inflammation via the regulation of inflammatory cytokines, (ii) promote the proliferation phase by enhancing angiogenesis and proliferation of fibroblast, and (iii) homeostasis of the remodeling phase by regulating collagen metabolisms. Therefore, it is worth noting that YaSP can be emphasized as a potential candidate for wound treatment, including diabetic wounds. However, its efficacy in the treatment of chronic wounds or infected wounds in both animal models and diabetic patients is urgently required.

## Data Availability

The original contributions presented in the study are included in the article/Supplementary Material, further inquiries can be directed to the corresponding authors.

## Abbreviations

COX-2, Cyclooxygenase-2; ECM , Extracellular matrix; GK rat, Goto-Kakizaki rat; HPLC, High-performance liquid chromatography; ICH, International Council for Harmonisation of Technical Requirements for Pharmaceuticals for Human Use; IL-1β, Interleukin-1β; iNOS, Inducible nitric oxide synthase; LC-MS/MS, Liquid chromatography-tandem mass spectrometry; LMICs, Low-to middle-income countries; MDA, Malondialdehyde; MPO, Myeloperoxidase; NO, Nitric oxide; PDII, The primary dermal irritation index; PGE_2_, Prostaglandin E_2_; T-YaSP, A traditional preparation of Ya-Samarn-Phlae; TGF-β1, Transforming growth factor-β1; TNF-⍺, Tumor necrosis factor-⍺; VEGF, Vascular endothelial growth factor; YaSP, An ointment containing T-YaSP.
