# Supplementary material for: Mechanisms underlying the wound healing and tissue regeneration properties of a novel gauze dressing impregnated with traditional herbal medicine (Ya-Samarn-Phlae) in type 2 diabetic Goto-Kakizaki (GK) rats
Source: Front Pharmacol. 2025 Apr 9;16:1574715. doi: 10.3389/fphar.2025.1574715 (PMC12015241; doi:10.3389/fphar.2025.1574715)
Supplement: Supplementary file 1 [file DataSheet1.docx]

**Supplementary TABLE 1**

Physicochemical parameters of herbal components of Ya-Samarn-Phlae (YaSP).

Parameters** *C. longa* *G. mangostana* *O. sativa A. catechu*

Foreign matter 0.06±0.03 (≤2.00) * 0.03±0.01 0.01±0.01 (≤2.00) ^#^ 0.01±0.02 (≤1.00) *

Loss on drying 7.10±0.16 (≤10.00) 3.49±0.04 NA 3.86±0.64 (≤12.00)

Total ash content 5.69±0.74 (≤8.00) 2.90±0.66 2.26±0.12 (≤6.00) 1.73±0.02 (≤2.00)

Acid-insoluble ash 0.19±0.01 (≤1.00) 0.22±0.03 0.14±0.03 (≤5.00) 0.14±0.01 (≤1.00)

Ethanol-soluble extractive 24.09±0.54 (≥10.00) 11.97±0.41 4.08±0.47 (≥1.00) 26.28±1.71 (≥25.00)

Water-soluble extractive 27.99±1.08 (≥9.00) 20.01±0.28 3.38±0.05 (≥1.00) 24.75±0.09 (≥10.00)

Hexane-soluble extractive 11.74±0.91 (≥5.00) NA NA NA

Tannin content NA NA NA 49.91±0.93 (≥24.00)

Volatile oil content 7.33±0.58 (≥6.00) NA NA NA

Curcuminoid content 6.69±0.43 (≥5.00) NA NA NA

* Standard parameters described in Thai Herbal Pharmacopoeia; # Standard parameters described in Ayurvedic pharmacopeia of India.

** With exception of the volatile oil content expressed as %v/w, all parameters were presented as %w/w.
NA; Not applicable

**Supplementary TABLE 2**

The viscosity quantification of the ointment base for Ya-Samarn-Phlae tulle-gras dressings (YaSP)

Formula No. Composition of coating materials (ratio; w/w) Viscosity of coated bases

White soft paraffin Hard paraffin T-YaSP (cP×10^3^)

WC1 1 - 4 114± 5.292

WC2 1 - 3 233± 3.055

WC3 3 - 7 309± 7.572

WC4 1 - 2 375± 4.163

WC5 2 - 3 448± 5.292

WC6 1 - 1 590± 6.000

WC7 4 - 3 629± 5.033

WC8 3 - 2 683± 6.110

WC9 2 - 1 755± 4.619

WC10 7 - 3 824± 6.000

WC11 3 - 1 885± 3.055

WC 12 4 - 1 1105± 6.429

WC13 9 - 1 1273± 3.055

HC1 - 1 3 4720± 60.000

HC2 - 1 4 407± 3.060

HC3 - 1 5 212± 9.170

HC4 - 1 6 127± 6.430

HC5 - 1 7 107± 6.430

HC6 - 1 8 81± 4.160

HC7 - 1 9 66± 4.000

**Supplementary TABLE 2**

The viscosity quantification of the ointment base for Ya-Samarn-Phlae tulle-gras dressings (YaSP)

Formula No. Composition of coating materials (ratio; w/w) Viscosity of coated bases

White soft paraffin Hard paraffin T-YaSP (cP×10^3^)

HC8 - 1 3.5 1473± 30.550

HC9 - 1 3.6 1245± 4.163

HC10 - 1 3.7 1094± 5.291

HC 11 - 1 3.8 897± 4.163

HC12 - 1 3.9 682± 4.000

WHC1 1 0.5 3.8 1504± 4.666

WHC2 5 0.5 3.8 1273± 3.055

WHC3 6 0.5 3.8 1084± 3.060

WHC4 7 0.5 3.8 1042± 4.056

The viscosity quantification of the ointment base for sofra-tulle®, a reference product, was found to be 894± 2.000 cPx10^3^.

**Supplementary TABLE 3**

Impact of multiple freeze-thaw cycles (FT; *n*=5) on the viscosity property, content of metabolites (curcumin and α-mangostin), and malonaldehyde level of the ointment base for Ya-Samarn-Phlae tulle-gras dressings (YaSP)

Parameters (mean ± SD) Before FT After FT

Content of YaSP (g) 6.82±0.29 6.21±0.18

Viscosity (x10^3^ cP) 890.67±3.06 894.00±4.00

Malonaldehyde (mg/kg) < 0.002* 0.49±0.0016

Alpha-mangostin (mg/g) 0.36±0.01 0.35±0.01

Curcumin (mg/g) 0.47±0.02 0.43±0.03

*The limit of detection of malonaldehyde is 0.002 mg /kg

**Supplementary TABLE 4**

Effect of long-term storage on chemical profiles of the ointment base for Ya-Samarn-Phlae tulle-gras dressings (YaSP)

Identification compounds Math score* Storage time (months)

Initial 3 6

1 Alpha-mangostin 96.29 + + +

2 Bisdemethoxycurcumin 96.21 + + -

3 Curcumin 95.93 + + +

4 Dihydrorotenone 49.40 + + +

5 3,5-Pyridinedicarboxylic acid, 1,4- 49.10 + - -

dihydro-2,6-dimethyl-4-(3-nitrophenyl)-,

carboxymethyl methyl ester

6 Methuyl tanshinonate 48.99 + + +

7 Stigmatellin Y 48.66 + - -

8 6,8-Dihydroxy-1,7-diprenylxanthone- 48.59 + + -

2-carboxylic acid

9 2,3-Dihydroabscisic alcohol 48.29 + - -

10 Glyinflanin H 48.20 + - -

11 Verimol C 48.04 + - -

12 Myristicanol B 47.93 + - -

13 Demethoxycurcumin 46.73 + + +

14 Mangostinone 34.94 + - -

* Math score at initial. +; Present -; Absent

**Supplementary TABLE 5**

Dermal irritation study of the ointment base for Ya-Samarn-Phlae tulle-gras dressings (YaSP) at different time intervals in the rabbits.

Rabbit No. Time after removal of patches (h)

1 24 48 72

1 1/0 0/0 0/0 0/0

2 0/0 1/0 0/0 0/0

3 0/0 0/0 0/0 0/0

Total 1/0 1/0 0/0 0/0

Mean 0.3/0 0.3/0 0/0 0/0

Primary Dermal Irritation Index (PDII) classifications are described as follows: negligible: 0–0.4; slight irritation: 0.5–1.9; moderate irritation: 2–4.9; and severe irritation: 5–8. Primary Dermal Irritation Index (PDII) =2/ (4x3) = 0.17; Toxicity Category IV (non-to slightly irritating). Values are expressed as scores of erythema/edemas.


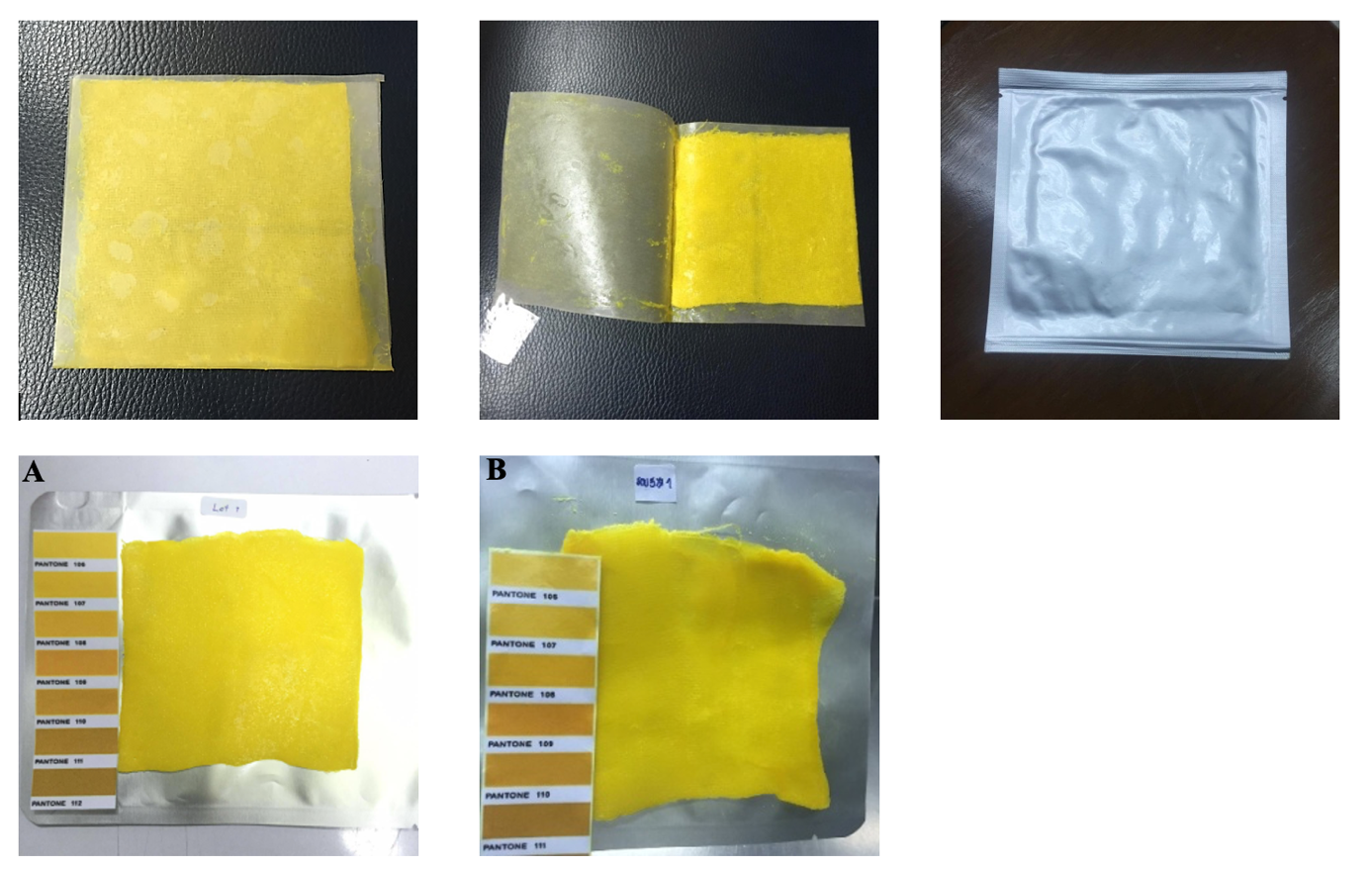


**Supplementary FIGURE 1** The appearance of tulle-gras wound dressings impregnated with the ointment base of Ya-Samarn-Phlae (upper panels) and the effect of multiple freeze-thaw cycles on their color (lower panels) was additionally observed. A and B represent the appearance of the tulle-gras wound dressings before and after being subjected to five freeze-thaw cycles at -39±2°C for 24 h and 45±2°C for 24 h.

**Supplementary FIGURE 2** The fasting blood glucose levels of the rats after being treated with ointment base for Ya-Samarn-Phlae tulle-gras dressings (YaSP) were recorded. The data represent the mean ± SEM of 5-6 animals per group.
